# Management of oroantral fistula with displacement of the root into sinus using buccal fat pad: a case report

**DOI:** 10.11604/pamj.2022.41.85.31368

**Published:** 2022-02-01

**Authors:** Soukaina Essaket, Yousra Zemmouri, Saliha Chbicheb

**Affiliations:** 1 Department of Oral Surgery, Consultation Center of Dental Treatment, Faculty of Dentistry, University Mohamed V, Rabat, Morocco

**Keywords:** Oroantral fistula, intrasinus root, buccal fat pad, case report

## Abstract

Oroantral fistula (OAF) refers to a permanent connection between oral cavity and maxillary sinus. The extraction of maxillary posterior teeth is the most common reason of OAF. Multiple techniques are available to repair this defect. The most used procedures involving local flaps include buccal flap, buccal fat pad (BFP), and palatal rotating flap. We here present an original technique of management of oroantral fistula with displacement of the root into sinus using BFP.

## Introduction

Oroantral communication (OAC) is an abnormal communication between the maxillary sinus and the oral cavity, which, if not treated, may progress to oroantral fistula (OAF) [1,2]. The oral and antral cavities have a permanent communication by means of a fibrous channel lined by epithelium. It may present a purulent discharge dripping from the fistula, especially when head is lowered, regurgitation of liquid food into the nose with nasal discharge, and leakage of air through the fistula into the mouth due to nose blowing, development of an antral polyp. Sinus infection is responsible for facial pain and headache [3,4].

The most common cause of this complication is the extraction of posterior maxillary teeth due to their root´s proximity to the maxillary sinus and thin antral floor in this area. However, it can also be caused by a pathological condition such as osteonecrosis, cyst, tumor or by iatrogenic procedures such as implant surgery, cyst and tumor enucleation, orthognathic surgery [5,6]. Surgical techniques for OAF closure include autogenous soft tissue and bone grafting, allograft, xenograft, synthetic graft materials, and other techniques as tooth transplantation. Flap surgery techniques can be categorized into local and distant flaps, for example tongue flap. The most used are local flap procedures; they include buccal flap, buccal fat pad (BFP), and palatal rotating flap [7].

Before choosing a technique, many aspects should be considered, including the size of the fistula, the time of diagnosis, and the presence of sinus infection. In general, OAF closure within 48 hours of onset is recommended to avoid sinus infection. Spontaneous closure of the fistula may occur if the fistula is smaller than 3 mm in diameter. If the fistula measures less than 5 mm, suturing of the surrounding gingiva may be enough. Flap surgery is usually recommended if the entrance of the fistula is larger than 5 mm [8,9]. This study presents oroantral fistula management based on buccal fat pad flap with the use of alveolar crestal approach for the removal of root from the sinus instead of another surgical intervention such as endoscopic sinus surgery or the Caldwell-Luc procedure.

## Patient and observation

**Patient information:** a 52-year-old female presented with a chief complaint of nasal regurgitation, pain and halitosis. Relevant dental history revealed extraction of maxillary left second molar 7 days before.

**Figure 1:**
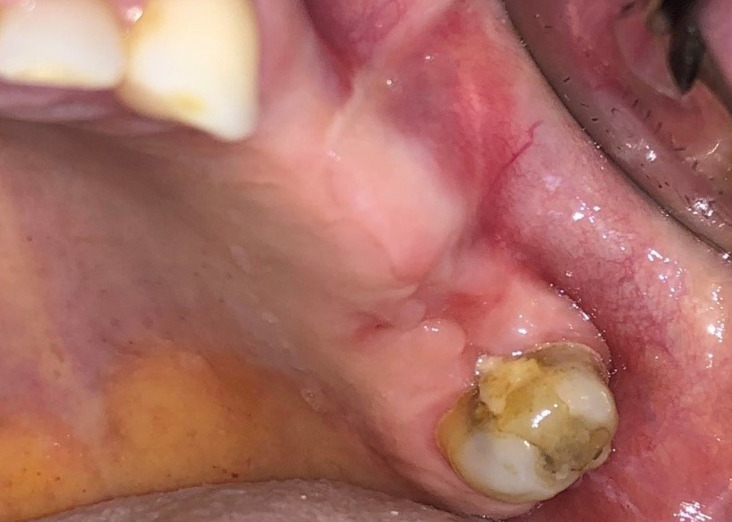
intraoral view of oroantral fistula

**Figure 2:**
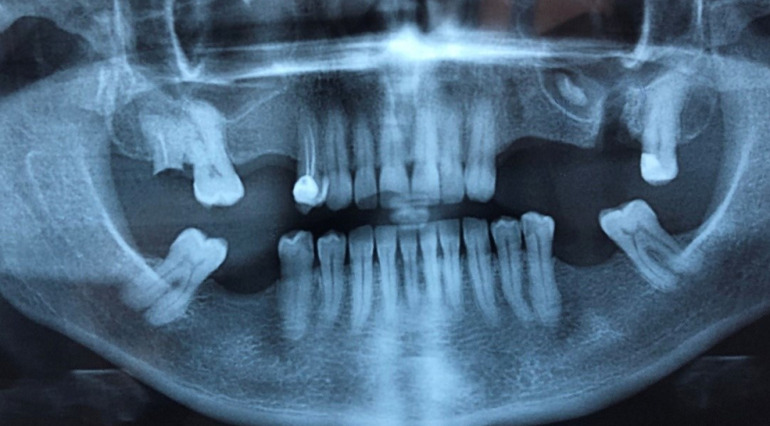
panoramic X-ray showing the oroantral fistula with intrasinus displacement of root of maxillary left second molar in the sinus

**Diagnosis:** clinical and radiographic investigation was done to confirm oroantral fistula with displacement of root of maxillary left second molar in the sinus (Figure 1, Figure 2). The patient was made aware of condition, treatment plan and all risk of surgery and received preoperative medications.

**Figure 3:**
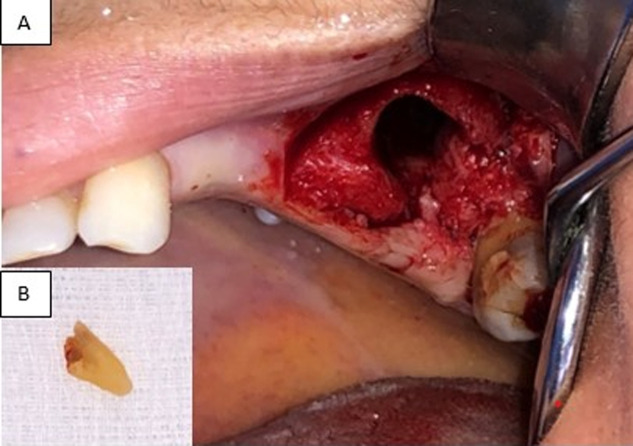
A) intraoral view of the mucoperiosteal flap; B) the extraction of the intrasinus root using the alveolar crestal approach

**Figure 4:**
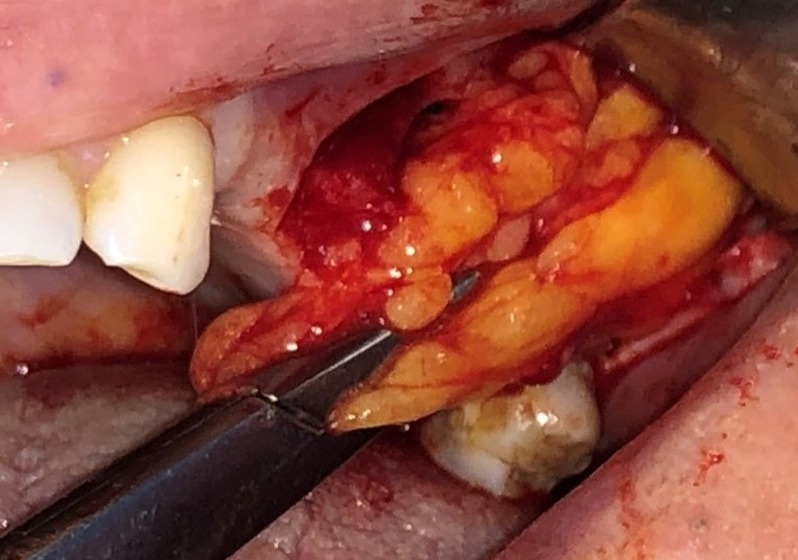
intraoral view of the traction of the buccal fat pad to the OAF site

**Figure 5:**
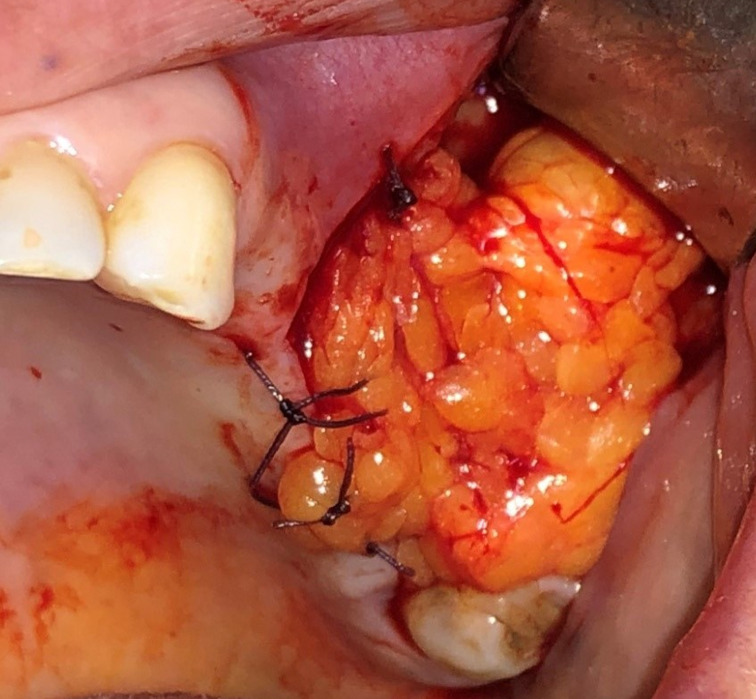
intraoral view of the suture of the fat pad

**Figure 6:**
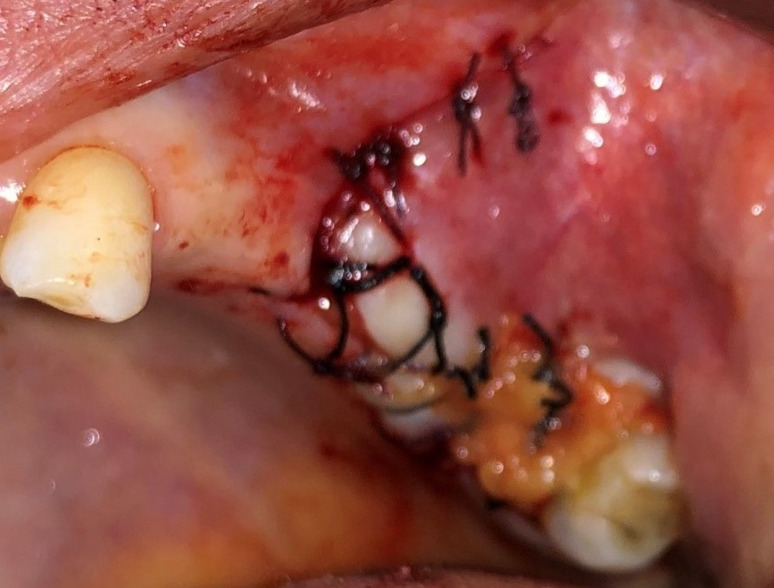
intraoral view of the suture of the mucoperiosteal flap

**Therapeutic interventions:** mucoperiosteal flap with a trapezoidal shape and two vertical releasing incisions were elevated under local anesthesia. Root removal using alveolar crestal approach was performed (Figure 3). The socket and the sinus were irrigated with physiological serum. A simple incision through the fascial envelope of the buccal fat pad was performed, and traction of the fat to the OAF site was done (Figure 4). The buccal fat pad was sutured without tension (Figure 5). The mucoperiosteal flap was returned and sutured (Figure 6). Postoperative instructions and medication were given and suture removal was scheduled after 10 days.

**Follow-up:** healing was good and uneventful with no nasal regurgitation and pain during the 6-months follow-up.

## Discussion

Buccal fat pad is an encapsulated mass of specialized fatty tissue, the volume of which varies throughout life. Buccal fat pad consists of a body and four extensions. The body is located behind the zygomatic arch and divided into 3 lobes. The extensions are the buccal, the pterygoid, and the superficial and the deep temporal [10]. Similarly to our case, BFP technique is based on the realization of a mucoperiost flap and fascial envelope incision. This incision opens the space of the buccal fat pad. The extension of buccal fat pad is advanced carefully toward to OAC, respecting a large pedicule. It is sutured, without tension to avoid necrosis of the flap on the de-epithelized margins of the fistula. The mucoperiosteal flap is then repositioned and sutured [11].

The presence of maxillary sinusitis, epithelialization of the fistula, dental apical abscess, osteitis, osteomyelitis of the communication´s margins, dental cysts, foreign bodies or tumors can prevent spontaneous healing and results in chronic fistula formation. Thus, elimination of the maxillary sinus pathology is essential for successful treatment of OAF. This justified, in our case, the removal of the root before the closure of the fistula [5,12]. In patients with acute sinus infection, amoxicillin/clavulanate 1 g/125 mg three times per day for 10 to 14 days, nasal decongestants, and non-steroidal anti-inflammatory drugs can be prescribed to manage the infection, according to the recent literature. Routine sinus irrigation could be helpful alongside the use of these medications. Meanwhile, patients with chronic sinus disease require surgery such as endoscopic sinus surgery or Caldwell-Luc operation [8].

Postoperative instructions are very important and must be followed. They comprise: maintaining oral care, soft food diet, use of analgesics and nasal decongestants. The patient should avoid nose blowing, sneezing with the mouth closed, and vigorous sports [2]. Buccal fat pad is a simple technique, easy to perform and associated with a low complication rate. It shows high success rate because of the rich vascularity of fat pad. Indeed, in a recent study, this was the most successful flap procedure (98.3%), followed by buccal flap (89.8%), and palatal flap (85.7%). This technique has many benefits, such as covering large defects without loss of sulcus depth [5,13,14].

Buccal fat pad requires a very careful manipulation. This technique is not recommended to patients with a history of radiation therapy because the size and mobility of fat pad are affected. Buccal fat pad has also some limitations; the closure of large defects requires traction of a greater portion, which can cause aesthetic depression of the cheek. The most common complications include persistent OAF and mouth opening limitation [8,14].

## Conclusion

OAF is a permanent communication between oral cavity and maxillary sinus. The extraction of maxillary posterior teeth is the most common reason of OAF. Multiple techniques are available to repair this defect. Based on our experience, the use of buccal fat pad flap is a simple technique with a high success rate.
